# *E. coli* “Stablelabel” S30 lysate for optimized cell-free NMR sample preparation

**DOI:** 10.1007/s10858-023-00417-4

**Published:** 2023-06-13

**Authors:** Roman Levin, Frank Löhr, Betül Karakoc, Roman Lichtenecker, Volker Dötsch, Frank Bernhard

**Affiliations:** 1 Institute of Biophysical Chemistry and Center for Biomolecular Magnetic Resonance, Goethe University, 60438 Frankfurt, Germany; 2 Institute of Organic Chemistry, University of Vienna, 1090 Vienna, Austria; 3 MAG-LAB, 1030 Vienna, Austria

**Keywords:** Cell-free expression, Stable isotope scrambling, Protein labeling, S30 lysate, NMR of membrane proteins, Metabolic engineering

## Abstract

Cell-free (CF) synthesis with highly productive *E. coli* lysates is a convenient method to produce labeled proteins for NMR studies. Despite reduced metabolic activity in CF lysates, a certain scrambling of supplied isotope labels is still notable. Most problematic are conversions of ^15^N labels of the amino acids L-Asp, L-Asn, L-Gln, L-Glu and L-Ala, resulting in ambiguous NMR signals as well as in label dilution. Specific inhibitor cocktails suppress most undesired conversion reactions, while limited availability and potential side effects on CF system productivity need to be considered. As alternative route to address NMR label conversion in CF systems, we describe the generation of optimized *E. coli* lysates with reduced amino acid scrambling activity. Our strategy is based on the proteome blueprint of standardized CF S30 lysates of the *E. coli* strain A19. Identified lysate enzymes with suspected amino acid scrambling activity were eliminated by engineering corresponding single and cumulative chromosomal mutations in A19. CF lysates prepared from the mutants were analyzed for their CF protein synthesis efficiency and for residual scrambling activity. The A19 derivative “Stablelabel” containing the cumulative mutations *asnA, ansA/B, glnA, aspC* and *ilvE* yielded the most useful CF S30 lysates. We demonstrate the optimized NMR spectral complexity of selectively labeled proteins CF synthesized in “Stablelabel” lysates. By taking advantage of *ilvE* deletion in "Stablelabel", we further exemplify a new strategy for methyl group specific labeling of membrane proteins with the proton pump proteorhodopsin.

## Introduction

Cell-free (CF) expression reactions are open systems that allow precise definition of conditions and compound concentrations during protein synthesis. Complete control of the amino acid pool in combination with low reaction volumes make them particularly useful for selective and combinatorial amino acid labeling (Reckel et al. 2011; Su et al. 2011; Yokoyama et al. 2011; Apponyi et al. 2008; Laguerre et al. 2015; Kigawa et al. 1999). *E. coli* S30 lysates prepared by centrifugation at 30,000×*g* are the most standard core component of CF reactions (Foshag et al. 2018). Removal of several amino acid metabolic enzymes during S30 lysate preparation already reduces molecular conversion and increases the stability of expensive labeled amino acids during CF synthesis of proteins for NMR analysis (Laguerre et al. 2015; Foshag et al. 2018; Hoffmann et al. 2018). However, *E. coli* S30 lysates still display significant scrambling of some amino acids such as L-Asn, L-Asp, L-Gln and L-Glu (Yokoyama et al. 2011; Tonelli et al. 2012). Further problems are notable with membrane proteins which need to be incorporated into bicelles or lipid nanoparticles such as nanodiscs (Reckel et al. 2011; Laguerre et al. 2015). Due to the intrinsic low tumbling rates of membrane proteins, deuterated amino acids are preferred for labeling (Sattler and Fesik 1996; Löhr et al. 2003). However, several aminotransferases still present in *E. coli* S30 lysates cause deuterium-proton exchange at the Cα atoms. This results in double or even triple signals that further increase NMR spectra complexity and ambiguity, while reducing sensitivity. Furthermore, aminotransferases are contributing to ^15^N label scrambling between different amino acids.

**Fig. 1:**
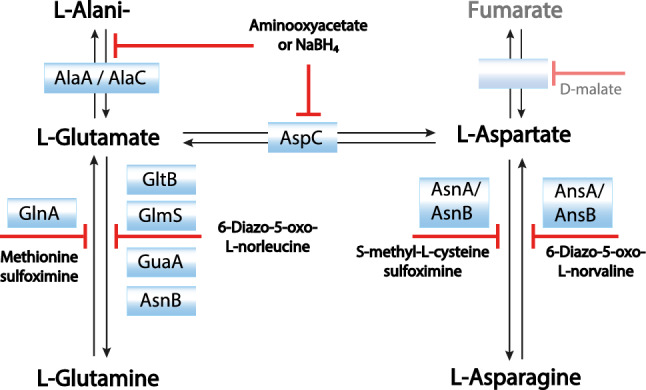
Action of inhibitors used to suppress amino acid conversions in *E. coli* S30 CF lysates

To address NMR label scrambling problems in CF systems, unselective inhibitors such as aminooxyacetate (AOA) or NaBH_4_ are in use to suppress transaminase activity (Su et al. 2011; Laguerre et al. 2015; Tonelli et al. 2012). AOA is mostly preferred as NaBH_4_ is toxic and potentially highly reactive with other CF reaction compounds. To gain control over amino acid conversions between L-Glu, L-Gln, L-Asp and L-Asn in CF reactions, numerous inhibitors were proposed and specific cocktails are used in some commercial CF expression kits (Fig. 1) (Yokoyama et al. 2011). Alternatively, the elimination of putative scrambling enzymes by genetic engineering can be considered. For conventional NMR sample preparation in intact *E. coli* cells, engineered derivatives containing single auxotrophic mutations and/or reduced transaminase activities were already reported (Lin et al. 2011; Waugh 1996). In CF lysates, the elimination of enzymes responsible for amino acid degradation such as SdaA, GdhA, TnaA and SpeA improved stability of some labeled amino acids during CF expression reactions (Michel-Reydellet et al. 2004). Scrambling of L-Glu to L-Gln was eliminated by *glnA* inactivation in the lysate source strain as a strategy to create in vitro L-Gln sensors (Soltani et al. 2021). Defined reconstituted CF systems such as the commercial PURE system are generally devoid of enzymes involved in amino acid metabolism, but hardly applicable for NMR sample preparation due to limited efficiency (Shimizu et al. 2001; Lavickova and Maerkl 2019).

The commonly used S30 lysates can be prepared from many different *E. coli* strains including *E. coli* K12 as well as BL21 derivatives. In addition, S30 lysate preparation protocols are highly diverse and thus, protein production efficiencies of various S30 lysate batches and their final proteome compositions may differ considerably. Depending on the preparation procedure, up to 50% of the initial *E. coli* proteome including many metabolic enzymes are removed. To obtain a reliable S30 lysate quality with a reproducible proteome composition, we have standardized the S30 lysate production procedure from *E. coli* A19 frequently used for CF membrane protein synthesis (Foshag et al. 2018; Klammt et al. 2004; Schwarz et al. 2007). The final A19 S30 lysate proteome was analyzed by detailed proteomics studies, and remaining amino acid converting enzymes causing label scrambling were identified (Foshag et al. 2018). In this study, we have systematically created individual and combinatorial chromosomal mutations in A19 genes encoding for these remaining scrambling enzymes in the standard S30 lysate. S30 lysates obtained from A19 variants were subsequently prepared and analyzed for NMR label scrambling as well as for CF protein production capacity. As result, we propose the A19 derivative “Stablelabel” containing combinatorial mutations of *asnA, ansA/B, glnA, aspC* and *ilvE* as source for efficient S30 lysates suitable for optimized CF NMR sample preparation. Benefits of “Stablelabel” S30 lysates over A19 S30 lysates were indicated by comparing residual activities in amino acid scrambling as well as in amino acid synthesis, potentially resulting in NMR label dilution. In addition, we exemplify a new approach to synthesize methyl group labeled NMR samples using “Stablelabel” S30 lysates with the proton pump proteorhodopsin.

## Materials and methods

All unlabeled amino acids used in this study were of analytical grade and purchased from Carl Roth (Germany) or Sigma-Aldrich (USA). All single ^15^N labeled amino acids as well as labeled α-ketoisovaleric acid (3-methyl-^13^C, 3, 4, 4, 4-D4) (KIV) were purchased from Cambridge Isotope Laboratories, Inc. (USA). Labeled 4-methyl-2-oxovaleric acid (4-methyl-^13^C, 3, 3, 4, 5, 5, 5-D6) (MOV) was synthesized as reported (Lichtenecker et al. 2015). 6-diazo-5-oxo-L-norleucine (DON), aminooxyacetate (AOA), and D-malate (DM) were obtained from Sigma-Aldrich (USA).

### Genetic engineering of* E. col*i A19

Gene deletions in A19 were performed according to Datsenko and Wanner (Datsenko and Wanner 2000) as adapted by the protocol of the Gene Bridges Quick and Easy™ gene deletion kit. FRT-PGK-gb2-neo-FRT cassettes with flanking regions homologous to the target genes were generated by PCR and generated kanamycin resistance (KanR) if integrated into the A19 chromosome. First, A19 cells were transformed with the pRed/ET plasmid and cultivated at 30 °C to an OD of approx. 0.6 in reaction tubes with punctured lids and shaking at 700 rpm. Expression of λ phage recombination proteins was induced by addition of arabinose to a final concentration of 0.3% (w/v) and incubated at 37 °C for 45 min. Subsequently, cells were cooled to 4 °C, harvested by centrifugation at 10,000×*g*, 4 °C for 1 min, washed three times with 1.5 mL ice-cold 10% (v/v) glycerol and harvested by centrifugation at 10,000×*g*, 4 °C for 1 min. The cell pellet was suspended in 40 µL 10% (v/v) glycerol solution and 1–2 µg linear FRT-PGK-gb2-neo-FRT cassette flanked by specific homology regions was added to the suspension before electroporation at 1450 V and ~ 5 ms pulse duration. Primers used for cassette generation are listed in Table 1. Subsequently, LB medium was added to the cells which were then incubated at 37 °C for another 3 h before being plated on kanamycin agar plates and incubated at 37 °C for 16–48 h. pRed/ET plasmid was cured during 37 °C incubation steps. Mutants were screened via colony PCR using KanR and gene locus specific primer pairs or primers annealing up-and downstream of the target gene (Table 1). Additionally, PCR products were purified and sequenced. For marker recovery, clones were transformed with 708-FLPe plasmid carrying FLP recombinase and plated on agar plates containing kanamycin and chloramphenicol. Clones were picked and grown in LB medium at 30 °C, to an OD of 0.6. A temperature shift to 37 °C induced the expression of FLP recombinase, which removed the marker from the gene locus via FRT recognition sites. FLP plasmid was cured during cultivation at 37 °C. Recovery was confirmed by PCR of the gene locus by subsequent PCR product purification and sequencing. Growth media were supplied with L-Gln or glucosamine if required for specific mutants. The "Stablabel" mutant containing mutations of *asnA*, *ansA*, *ansB*, *glnA*-*his*, *aspC* and *ilvE* was deposited in the German collection of microorganisms and cell cultures under the accession number DSM 116065.

**Table 1:** Primers used for *E. coli* A19 mutagenesis. “KO”-primers are used to generate a KanR cassette flanked by regions for homologous recombination

Primer name	Sequence
asnA-KO-fw	GCATGTGTGTCGGTTTTTGTTGCTTAATCATAAGCAACAGGACGCAGGAGAATTAACCCTCACTAAAGGGCG
asnA-KO-rev	GATACCGGGATGCGAAGCCGCCTGCTCAGACGCTGGCGGCGATAAATTATAATACGACTCACTATAGGGCTC
asnA-genscr-fw	GCCAAATTGTTTCGCCAG
asnA-genscr-rev	GGTTTGATGCCGATGCG
asnB-KO-fw	CAAGCAAACACAACAAGCAACAAATACCAGGTTAACGGAGAAGGTTATGTAATTAACCCTCACTAAAGGGCG
asnB-KO-rev	CCTCAATAACTGAAACAGCCCCGGATTTCACCGGGGCTGTTTCGCATTTCTAATACGACTCACTATAGGGCTC
asnB-genscr-fw	GCATCCGGCATGAACAAATCG
asnB-genscr-rev	GTTTGGTTAGGTTGCGAACAG
ansA-KO-fw	CTGCCTCACGTATATACTTTTGCTCTTTCGATATCATTCATATCAATATCAATTAACCCTCACTAAAGGGCG
ansA-KO-rev	CATTTTGTAAATCGACCAGTAACAGGGCGCGAGGGGGCATTACAGTCTCCTAATACGACTCACTATAGGGCTC
ansA-genscr-fw	CGTTGATGAACCGACCTTC
ansA-genscr-fw	CACGCAATAGTCAGTAGC
ansB-KO-fw	GCGATTAGTACTGATTGAAGATCTGCTGGATCTGCTGCGGATCTTTGGTTTG AATTAACCCTCACTAAAGGGCG
ansB-KO-rev	CAGAGCTAAGGGATAATGCGTAGCGTTCACGTAACTGGAGGAATGAAATGTAATACGACTCACTATAGGGCTC
ansB-genscr-fw	CGTTCCTTTACTGGTTAAC
ansB-genscr-rev	CAAACTTGATGCGCAGC
gltB-KO-fw	ATGACACGCAAACCCCGTCGCCACGCTCTTTCTGTGCCCGTGCGCAGCGGAATTAACCCTCACTAAAGGGCG
gltB-KO-rev	TTACTGCGCCTGCACGCGCAACTCTGCTGCGCTACGACTACGGTGACCCATAATACGACTCACTATAGGGCTC
gltB-genscr-fw	CCTTTTGCCCATAACGAC
gltB-genscr-rev	GGATGTAGTTGTGTACCGG
glnA-His-fw	GCGTGCGTATGACTCCGCATCCGGTAGAGTTTGAGCTGTACTACAGCGTCCATCACCATCACCATCACTAAAATTAACCCTCACTAAAGGGCG
glnA-His-rev	GATCCCAGGCCTGCCAGAGACAGGCGAAAAGTTTCCACGGCAACTAAAACACTAATACGACTCACTATAGGGCTC
glnA-genscr-fw	GTGATCGCTTTCACGGAG
glnA-genscr-rev	GTCATCTTCTTCGCGACG
glmS-KO-fw	CGTCTGGAATACCGCGGATATGACTCTGCCGGTCTGGCCGTTGTTGATGCAATTAACCCTCACTAAAGGGC
glmS-KO-rev	CACGCGCGCGAACTTCTTCAATGTTGGATTTCAGTTTTTCCAGCAATTCTAATACGACTCACTATAGGGCTC
glmS-genscr-fw	GCAACTGCTGGCTG
glmS-genscr-rev	GCGTCAACAGGTACAG
aspC-KO-fw	GTTACCCTGATAGCGGACTTCCCTTCTGTAACCATAATGGAACCTCGTCATAATTAACCCTCACTAAAGGGCG
aspC-KO-rev	GCTTTTCAGCGGGCTTCATTGTTTTTAATGCTTACAGCACTGCCACAATCGTAATACGACTCACTATAGGGCTC
aspC-genscr-fw	GCTGTGGGTATCGTTTACCAG
aspC-genscr-rev	GGATTTCTGGCAAAGTGCG
ilvE-KO-fw	GAGCACAACCACATCACAACAAATCCGCGCCTGAGCGCAAAAGGAATATAAAAAATTAACCCTCACTAAAGGGCG
ilvE-KO-rev	GTATTTATTGATTAACTTGATCTAACCAGCCCCATTTATCTTCGGTTTCGCCTAATACGACTCACTATAGGGCTC
ilvE-genscr-fw	CTTCGGCATCCATGGC
ilvE-genscr-rev	CGCAGTTAGAGATGCAGAC
KanR-screen-fw	GTATCCATCATGGCTGATG

“genscr”-primers are used for colony PCR screening and amplification of fragments. Blue and red sequences indicate upstream and downstream HR regions of the KO cassettes, respectively

### Preparation of S30 CF lysates

To produce lysates for CF expression, *E. coli* A19 cells were grown in 10 L YPTG (10 g/L yeast extract, 16 g/L tryptone, 5 g/L NaCl, 0.1 M glucose, 4.4 mM KH_2_PO_4_, 8 mM K_2_HPO_4_) tempered to 37 °C, 500 rpm and high aeration corresponding to ~ 3 reactor volumes of air per minute. Cells were grown until the culture reached an OD_600_ of 3.6–4.2 and then cooled down to 19 °C within 20 min. Subsequently, cells were harvested at 4500×*g*, 4 °C for 20 min and washed three times by centrifugation and suspension in 300 mL S30 A buffer (10 mM Tris–HCl, pH 8.2, 14 mM Mg(OAc)_2_, 60 mM KCl, 6 mM 2-mercaptoethanol). After washing, the pellet was suspended in 1.1 volumes of the pellet weight (mL = g) of S30 B buffer (10 mM Tris–HCl, pH 8.2, 14 mM Mg(OAc)_2_, 60 mM KOAc, 1 mM DTT and 1 × cOmplete protease inhibitor). Subsequently, the cells were lysed using a single French press passage at 1000 psig. The lysate was then centrifuged twice at 30,000×*g*, 4 °C for 30 min and the supernatant was subjected to a heat-step at 42 °C for 45 min. The lysate was then dialyzed in dialysis tubes of 10 MWCO against 5 L S30C buffer (10 mM Tris–acetate, pH 8.0, 14 mM Mg(OAc)_2_, 60 mM KOAc, 0.5 mM DTT) for 3 h at 4 °C and then for another 16 h at 4 °C in fresh 5 L S30C buffer. After dialysis, precipitate was removed by centrifugation at 30,000×*g*, 4 °C for 30 min.

### Recombinant expression of branched chain amino acid aminotransferase IlvE

IlvE was expressed in *E. coli* BL21 Star express cells as described previously (Lazarova et al. 2018). Briefly, 100 mL LB medium with 100 µg/mL ampicillin in a 0.5 L baffled shaking flask was inoculated with a single colony of freshly transformed cells and incubated at 37 °C, 180 rpm for 16 h. Then 1 L LB medium with 100 µg/mL ampicillin in a 2 L baffled shaking flask was inoculated with 20 mL of the preculture and incubated at 37 °C, 180 rpm until OD_600_ 0.6–0.9 was reached and 1 mM IPTG was added. The cells were cultivated for another 4 h under the same conditions before they were harvested, washed with IlvE buffer (100 mM Tris–HCl, pH 8.0, 100 mM NaCl), suspended in the same buffer with protease inhibitor cocktail and disrupted by sonication. The crude lysate was centrifuged at 30,000×*g* at 4 °C for 30 min, passed through a 0.45 µm filter and applied on a 5 mL HiTrap IMAC column previously equilibrated with IlvE buffer with 20 mM imidazole. The bound protein was then washed with another 15 CV of the same buffer and eluted with 4 CV IlvE buffer with 300 mM imidazole. The eluate was dialyzed against the 100-fold volume of IlvE buffer, concentrated using 10 kDa MWCO ultrafiltration devices and glycerol was added to a final concentration of 50% (v/v). The concentration of the protein stock was 10 mg/mL. The aliquoted protein solution was flash frozen in liquid nitrogen until further use.

### Amino acid conversion of KIV and MOV

Conversion of KIV (3-methyl-^13^C, 3, 4, 4, 4-D4) to L-Val(4-^13^C, 2, 3, 4, 4, 4-D5) and MOV (4-methyl-^13^C, 3, 3, 4, 5, 5, 5-D6) to L-Leu (5-^13^C, 2, 3, 3, 4, 5, 5, 5-D7) was carried out in 10 mL conversion mixture and in D_2_O. Substance amounts to yield final concentrations of 100 mM for Tris and NaCl, 350 mM for L-Glu and 70 mM for KIV or MOV were weighed. The components were dissolved together in 9 mL D_2_O and pH was adjusted to 7.5 with 2 M NaOH in D_2_O. Then the mixtures were completed with 1 mL of 10 mg/mL IlvE solution to yield a final concentration of 1 mg/mL. The mixtures were incubated at 30 °C, 180 rpm for 16 h and passed through 10 kDa MWCO ultrafiltration devices to remove IlvE. The KIV or MOV permeates were then used instead of L-Val or L-Leu in CF expression reactions.

### Cell-free protein expression

All proteins in this study were produced using S30 lysates prepared from *E. coli* K12 A19 and its indicated derivatives (Schwarz et al. 2007; Falkinham and Clark 1974). Briefly, expression was carried out in a two compartment CF expression configuration with a reaction mixture (RM) volume of 60 µL and a 13-fold feeding mixture (FM) volume. The compartments were separated by a 12–14 MWCO dialysis membrane. In a standard CF reaction, both compartments contained 1 mM of all 20 amino acids, an additional 1 mM each of L-Arg, L-Cys, L-Trp, L-Met, L-Asp and L-Glu, 20 mM acetylphosphate, 20 mM phosphoenolpyruvate, 2 mM dithiothreit, 0.21 mM folinic acid, 1 × cOmplete protease inhibitor, 100 mM HEPES–KOH, pH 8.0, 800 µM EDTA, 18 mM MgOAc and 170 mM KOAc. The RM additionally contained 0.3 U/µL RNasin, 0.5 mg/ml tRNA from *E. coli* MRE 600, 0.04 mg/ml pyruvate kinase, 0.35% (v/v) S30 lysate and 15 ng/µL template of either pET21a-GFP-6xHis, pIVEX2.3d-proteorhodopsin-6xHis or pIVEX2.3d-6xHis-CypD, respectively. Analytical scale screening reactions were conducted in customized reaction containers holding the RM and placed in cavities of 24-well microplates filled with 775 µl of FM. Expression of NMR samples was conducted with 3–6 mL RM and 15-fold FM in commercial Slide-A-Lyzer devices. Expression was carried out at 30 °C for 16 h at 180 rpm axial agitation. Subsequently, the RM was harvested and centrifuged at 18,000×*g*, 4 °C for 10 min.

### Labeling and purification of his-tagged CypD

Truncated CypD (*△43-207*) was CF expressed from pIVEX2.1d-6xHis-CypD plasmid and IMAC purified via N-terminal 6xHis-tag as described previously (Hein et al. 2016). Briefly, 5 mL IMAC Sepharose 6 Fast Flow resin was used and purification was performed in 50 mM HEPES, pH 7.5, 150 mM NaCl with 20 mM imidazole in the washing buffer and 400 mM imidazole in the elution buffer. After elution, CypD was dialyzed against 50 mM sodium phosphate, pH 7.0, 0.5 mM DTT for 12–16 h and concentrated using 10 kDa MWCO ultrafiltration devices. Samples were supplemented with 100 µg/mL streptomycin, 1 × cOmplete protease inhibitor, 0.15 mM DSS and 5% D_2_O, and had a final protein concentration of 100–150 µM. Labeling was carried out using one of the ^15^N labeled amino acids L-Glu, L-Gln, L-Asp and L-Asn, respectively, while the other 19 amino acids were non-labeled. Inhibitors aminooxyacetate (AOA) and 6-diazo-5-oxo-norleucine (DON) were applied in some reactions at concentrations of 20 and 5 mM, respectively.

### Labeling and purification of his-tagged proteorhodopsin

Proteorhodopsin (PR) was expressed and purified using a modified protocol previously published (Reckel et al. 2011). PR was CF expressed from pIVEX2.3d-proteorhodopsin-6×His plasmid in the presence of 0.4% (w/v) glyco-diosgenin (GDN) and 0.1% (w/v) diC7PC and purified via C-terminal 6×His-tag. PR methyl-labeled samples were CF synthesized in presence of 19 unlabeled amino acids and either 1 mM L-Val (4-^13^C, 2, 3, 4, 4, 4,-D5) converted from KIV (3-methyl-^13^C, 3, 4, 4, 4-D4) or 0.5 mM L-Leu (5-^13^C, 2, 3, 3, 4, 5, 5, 5-D7) converted from MOV (4-methyl-^13^C, 3, 3, 4, 5, 5, 5-D6). After harvesting, the samples were applied on an IMAC column equilibrated with NMR buffer (25 mM Na-acetate, pH 5.0, with 0.1% (w/v) diC7PC). The bound protein was then washed with 15 CV NMR buffer with 20 mM imidazole to remove impurities and GDN. The sample was eluted with NMR buffer containing 300 mM imidazole. The eluate was then concentrated to a volume of 1 mL using 10 MWCO centriprep ultrafiltration devices. The buffer was next exchanged on a PD Miditrap G25 column to NMR buffer without imidazole and concentrated again using 10 MWCO centriprep ultrafiltration devices. The final samples also contained 100 µg/mL streptomycin, 1 × complete protease inhibitor, 0.15 mM Sodium trimethylsilylpropanesulfonate and 5% D_2_O.

### NMR measurements

All [^15^N, ^1^H]-TROSY spectra of CypD were acquired at sample temperatures of 303 K on Bruker 600 MHz (Avance II), 700 MHz (Avance III HD), 800 MHz (Avance III HD), 900 MHz (Avance Neo) and 950 MHz (Avance III) NMR spectrometers, equipped with cryogenic ^1^H{^13^C/^15^N} triple-resonance probes. To take advantage of longitudinal ^1^H relaxation enhancement between scans, the Band-Selective Excitation Short-Transient (BEST) method was applied with proton pulses with a bandwidth of 4.8 ppm centered at 8.7 ppm (Farjon et al. 2009). The interscan delay was set to 0.3 s. All CypD samples had concentrations of 100–150 µM in a 5 mm NMR tube with 600 µL sample volumes.

Methyl ^1^H–^13^C correlation spectra of PR were acquired on a Bruker Avance Neo 950 MHz spectrometer equipped with a cryogenic probe at sample temperatures of 313 K. An XL-ALSOFAST-HMQC pulse sequence with the ^13^C–^1^H back-transfer period shortened to 2 ms combined with delayed decoupling was employed (Rößler et al. 2020a). The delay between scans was set to 0.7 s. Using gradient coherence selection, the sequence was suitable to eliminate otherwise strong *t*_1_-noise from detergent and acetate signals. The L-Val (4-^13^C, 2, 3, 4, 4, 4,-D5) labeled and the L-Leu (5-^13^C, 2, 3, 3, 4, 5, 5, 5-D7) labeled samples had concentrations of 80 µM and 120 µM, respectively, in 5 mm NMR tubes with 600 µL sample volume.

## Results

### A19 strain engineering for minimized S30 CF lysate scrambling activity

**Fig. 2:**
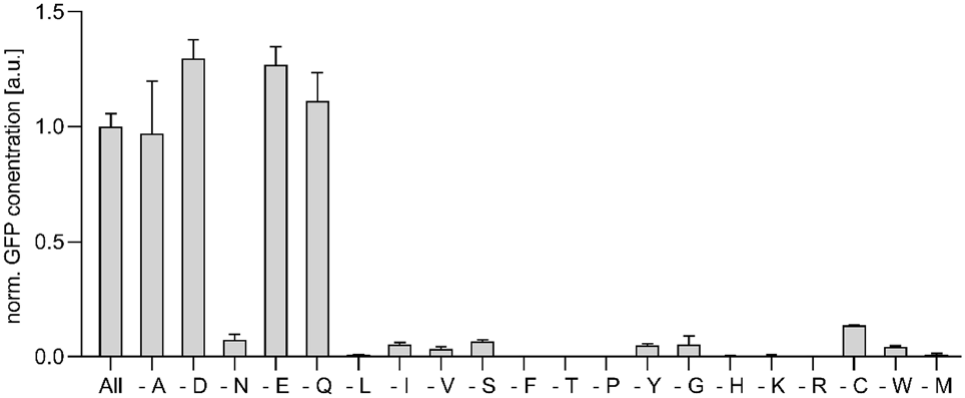
Analysis of amino acid scrambling via GFP expression. GFP expression in standard A19 S30 CF lysates carried out either in presence of all amino acids (all) or in absence of the indicated amino acids. Data normalized to GFP concentration of 3.5 mg/mL Quantification was carried out via fluorescence measurement in the RM after harvesting of the CF reaction. Bars represent means of expression yields obtained from at least 3 independent CF reactions

A considerable residual activity of amino acid conversions in A19 S30 CF lysates is notable for the amino acids L-Asp, L-Asn, L-Glu, L-Gln, and L-Ala. The absence of L-Asp, L-Glu, L-Gln, and L-Ala in CF expression reactions of the reporter protein GFP is completely compensated by internal conversions from other amino acids (Fig. 2). While only minor synthesis of L-Asn is observed, it is known to be the major source for conversion to L-Asp (Yokoyama et al. 2011). Some further internal formation in the lysate is noted for the amino acids L-Cys and L-Ser. Based on a previous proteomics analysis of standard A19 S30 CF lysates, nine residual enzymes most likely involved in the scrambling of L-Asp, L-Asn, L-Glu, L-Gln, and L-Ala were identified and the determined emPAI values could be taken as preliminary evidence for their abundance (Table 2) (Foshag et al. 2018). In addition, the transaminase IlvE converts branched chain amino acids and could cause label instability and backprotonation problems upon synthesis of deuterated samples. For all enzymes, construction of individual or sets of combinatorial knock-out mutations in A19 was approached. Even though extracellular asparaginase AnsB has not been detected by proteomics analysis, the corresponding gene was deleted as well due to the reported high substrate affinity of the AnsB enzyme if compared with the intracellular asparaginase AnsA. The *ansB* deletion may also become beneficial for future potential protocol modifications in S30 lysate preparation. A19 gene deletions were performed using the lambda recombination system as reported previously (Datsenko and Wanner 2000). Initially, we approached to remove GlnA posttranslationally from the S30 lysate by genetic modification with a terminal His-tag followed by subsequent IMAC purification. This would avoid the supply of L-Gln into growth media. However, we noticed that the genetic modification already abolished *glnA* expression and no GlnA-His was further detectable in S30 CF lysates by immunoblotting (data not shown).

**Table 2:** Enzymes with potential amino acid scrambling activity in standard A19 S30 lysates

Nr	Enzyme	EC	Gene	emPAI^a^
1	Asparagine synthase A	6.3.5.4	*asnA*	0.4 ± 0.2
2	Asparagine synthase (L-Gln hydrolyzing)	5.3.5.4	*asnB*	0.4 ± 0.3
3	Asparaginase	3.5.1.1	*ansA*	0.4 ± 0.2
4	Asparaginase II	3.5.1.1	*ansB*	-
5	Glutamate synthase [NADPH]	1.4.1.13	*gltB*	2.9 ± 1.1
6	Glutamine synthetase	6.3.1.2	*glnA*	6.2 ± 1.6
7	Glutamine-fructose-6-phosphate transaminase (GFAT)	2.6.1.16	*glmS*	5.9 ± 0.9
8	Aspartate aminotransferase	2.6.1.1	*aspC*	2.6 ± 0.6
9	Branched-chain-amino-acid transaminase (IlvE)	2.6.1.42	*ilvE*	0.4 ± 0.2
10	GMP synthetase (glutamine-hydrolyzing)	6.3.5.2	*guaA*	7.9 ± 1.0
11	Glutamate-pyruvate aminotransferase	2.6.1.2	*alaC*	0.1 ± 0.0

^a^emPAI values as determined (Foshag et al. 2018)

**Fig. 3:**
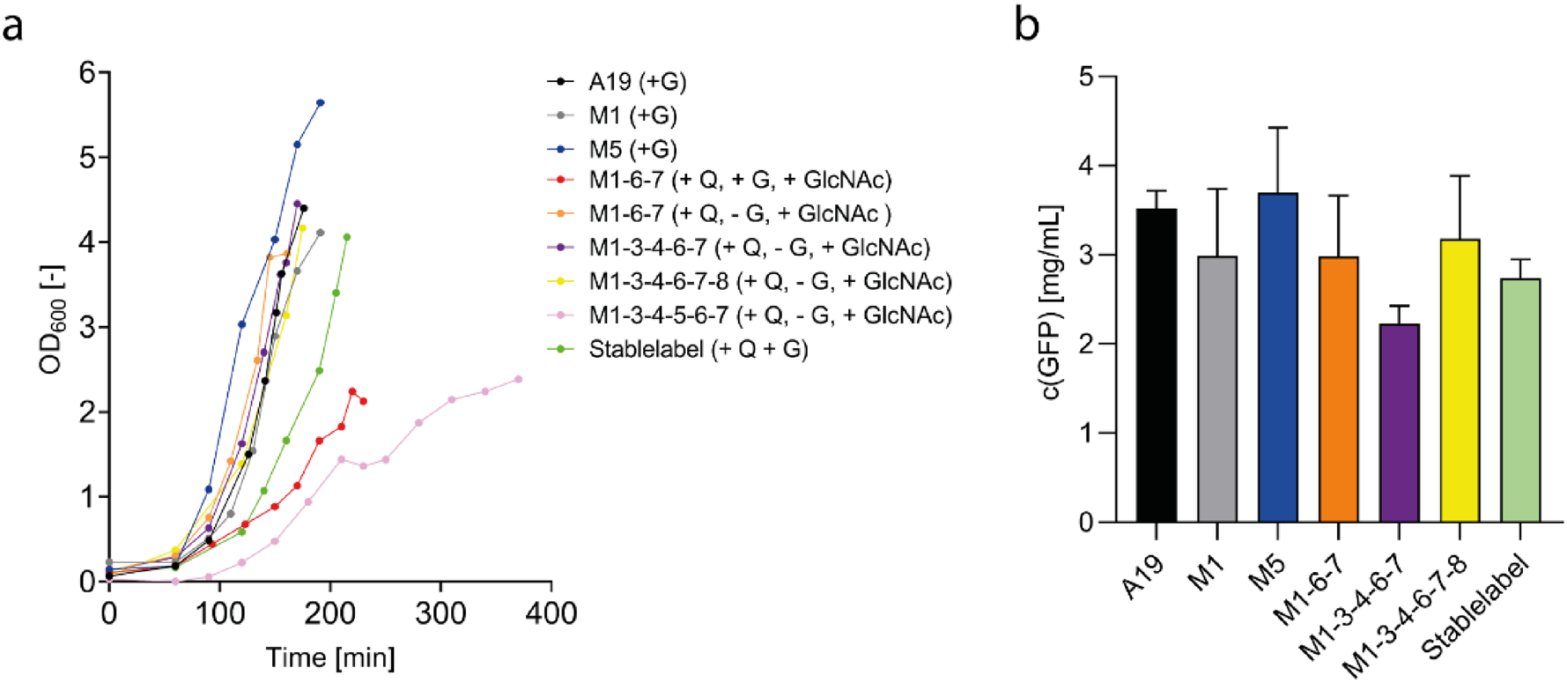
Growth kinetics and CF protein production performance of S30 lysates from selected A19 mutants. **A** Growth curves of *E. coli* A19 WT and mutants cultivated in a 15 L stirred-tank bioreactor in 10 L YPTG medium at 37 °C, 500 rpm. 1 g/L L-Gln was supplied to the culture medium to restore growth of *glnA* mutants (+ Q). Standard YPTG culture medium contained 0.1 M glucose (+ G), except for some *glmS* mutants such as M 1-3-4-5-6-7 (−G) where no glucose was added due to growth problems. Furthermore, *glmS* mutant cultures were supplied with additional 1 g/L N-acetyl D glucosamine (+ GlcNAc). **B** Extract performance was evaluated by expression of GFP under standard conditions. Quantification was carried out via GFP fluorescence measurement in the CF reaction mixture. Bars represent means of expression yields obtained from at least three independent CF reactions

Overall, 17 mutants were constructed in addition to two mutants of strain BW25113 obtained from the Keio collection (Baba et al. 2006) (Table 3). Attempts to delete *guaA* or *alaC* were not successful and the genes are thus probably essential for A19. Most of the obtained mutants showed growth curves comparable to that of wild type A19 (Fig. 3a). However, mutants containing knock-outs of *glnA* and/or *glmS* required medium supplementation with glutamine or D-glucosamine/N-acetylglucosamine (GlcNAc), respectively. Furthermore, the cumulative deletion *gltB* and *glmS* in strain M 1-3-4-5-6-7 significantly retarded the growth rate and only relatively low final OD_600_ values were obtained (Fig. 3a; Table 3). Another observation was that growth of *glmS* mutants only reached approx. OD_600_ = 2 when cultivated with 0.1 M glucose (Fig. 3a). Accordingly, it was previously suggested to omit glucose from culture medium upon cultivation of *glmS* mutants (Wu and Wu 1971). Mutant M 1-3-4-6-8-9 containing a combination of the most effective knock-outs was selected as further reference strain and renamed “Stablelabel” (DSM 116065). The strain did not contain a *glmS* mutation as conversion of L-Gln to L-Glu still remained and combined deletion of *glmS* with *gltB* led to poor growth and non-productive lysate*.* We therefore omitted *glmS* mutation to avoid the otherwise necessary supply of GlcNac or D-glucosamine into growth media. Inactivation of *ilvE* in “Stablelabel” was done to enable 3-methyl ^13^C-L-Val labeling based on the precursor KIV, which is especially interesting for membrane protein side chain labeling (Hoffmann et al. 2018). The final strain showed a slightly delayed growth curve if compared with the parental strain A19 probably mainly due to the deletion of the important transaminases AspC and IlvE (Fig. 3a).

**Table 3:** Constructed chromosomal mutants in strain A19

Number	1	2	3	4	5	6	7	8	9
Name	*asnA*	*asnB*	*ansA*	*ansB*	*gltB*	*glnA*	*glmS*	*aspC*	*ilvE*
K 3^a^			** + **						
K 4^a^				** + **					
M 1^a^	** + **								
M 5					** + **				
M 1-2	** + **	** + **							
M 1-2-3	** + **	** + **	** + **						
M 1-2-4	** + **	** + **		** + **					
M 1-2-4-5	** + **	** + **		** + **	** + **				
M 1-3-4-6-8	** + **		** + **	** + **		** + **		** + **	
M 1-2-4-5-6	** + **	** + **		** + **	** + **	** + **			
M 1-8	** + **							** + **	
M 1-2-5-8	** + **	** + **			** + **			** + **	
M 1-6^c^	** + **					** + **			
M 1-6-7^c^	** + **					** + **	** + **		
M 1-3-6-7^c^	** + **		** + **			** + **	** + **		
M 1-3-4-6-7^c^	** + **		** + **	** + **		** + **	** + **		
M 1-3-4-5-6-7^b, c^	** + **		** + **	** + **	** + **	** + **	** + **		
M 1-3-4-6-7-8^c^	** + **		** + **	** + **		** + **	** + **	** + **	
M 1-3-4-6-8-9^c^ “Stablelabel”	** + **		** + **	** + **		** + **		** + **	** + **

^a^Strains designated with a K resemble single knockout strains obtained from the Keio collection strain BW25113

^b^Poor or no strain proliferation

^c^A hisx6 tag was initially introduced to remove glutamine synthetase from the lysate. It was observed that introduction of hisx6 tag was sufficient to remove glutamine synthetase activity without interfering with lysate performance

CF S30 lysates were prepared from “Stablelabel” and from other selected mutants according to our standardized protocol and analyzed for their performance in CF expression reactions to synthesize the reporter protein GFP (Fig. 3b). Lysates from “Stablelabel” maintained considerable efficiency and synthesis yields of approx. 2.7 mg/mL GFP were obtained (Fig. 3b).

### NMR analysis of scrambling activity in “Stablelabel” S30 lysates

**Fig. 4:**
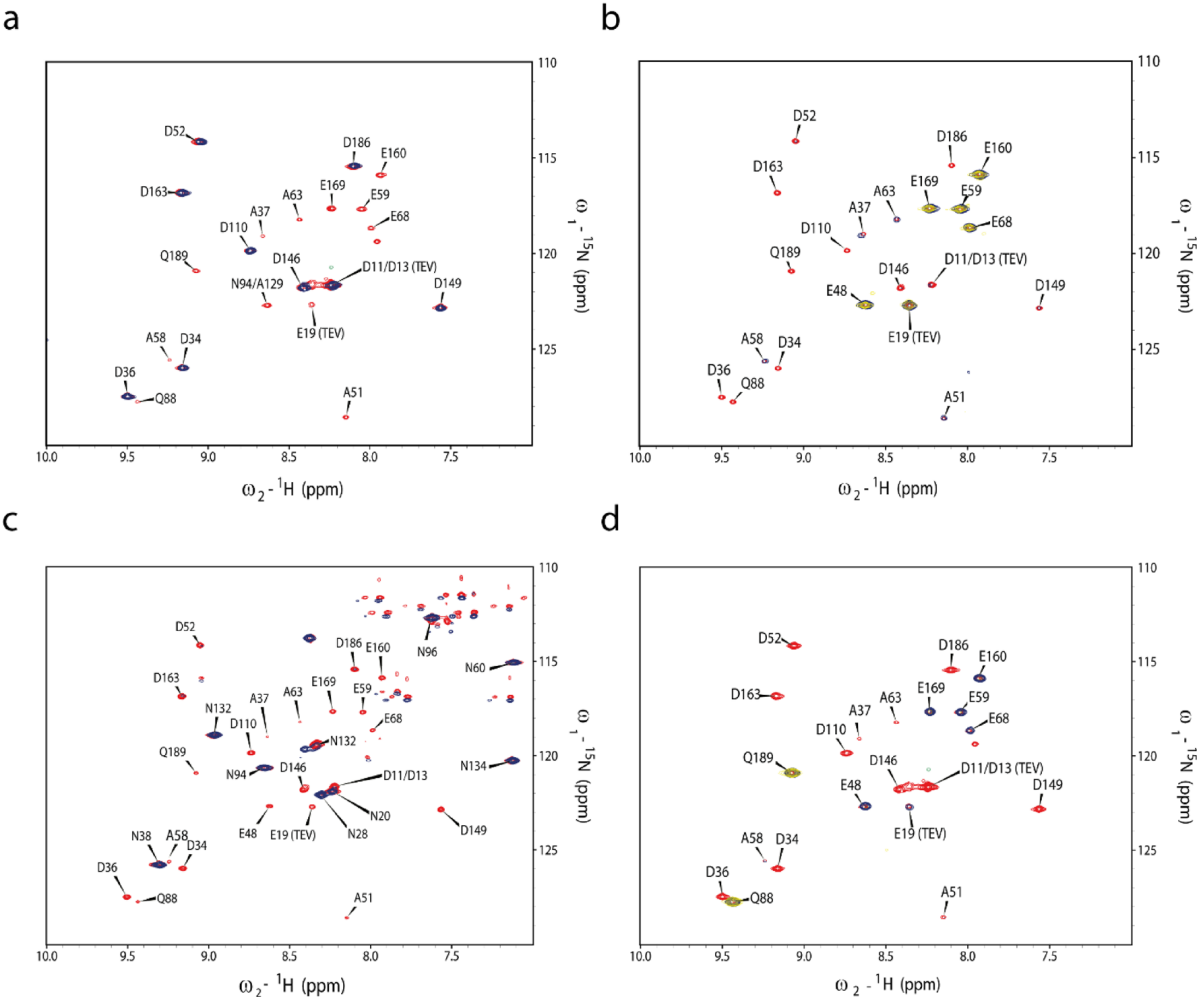
Selective CF labeling of CypD (43–207) with either A19 or “Stablelabel” S30 lysates. Spectra overlays illustrate results of CypD selective labeling with ^15^N L-Asp (**a**), ^15^N L-Glu (**b**), ^15^N L-Asn (**c**) or ^15^N L-Gln (**d**) using either A19 (red) or “Stablelabel” (blue) S30 lysates. The yellow spectrum in **b** was obtained after addition of 20 mM AOA in the CF reaction to suppress L-Glu to L-Ala label conversion. The yellow spectrum in **d** was obtained after incubation of CF lysate with 5 mM DON at RT for 1 h before CypD expression. [^15^N, ^1^H] BEST-TROSY spectra were measured on CypD samples of 100–150 µM concentration in sodium phosphate buffer pH 7.0 at 303 K. All A19 spectra were recorded on 900 or 950 MHz spectrometers while “Stablelabel” spectra were recorded on 600 or 700 MHz spectrometers

**Fig. 5:**
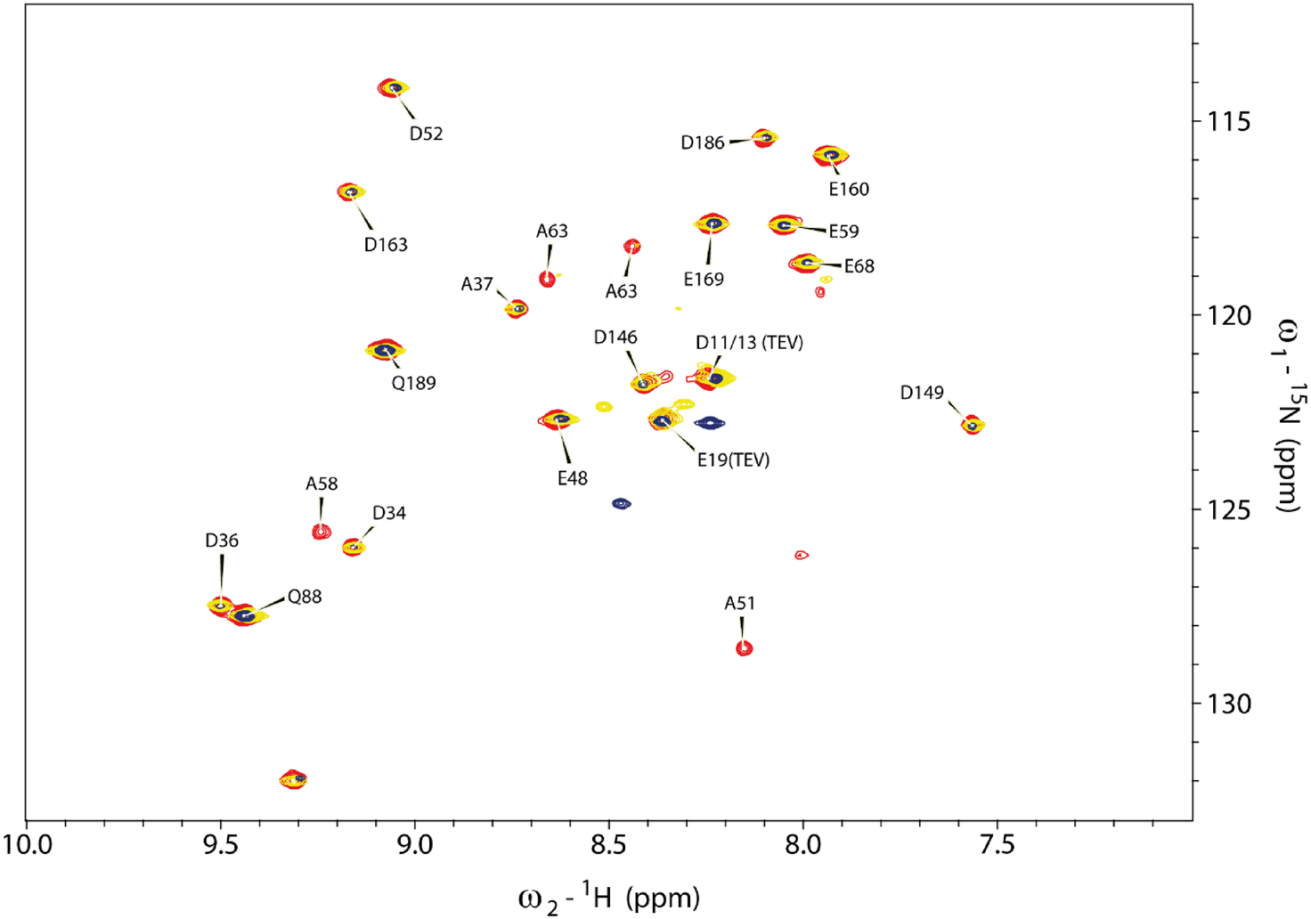
^15^N L-Gln labeling of CypD CF expressed with lysates of either A19 (red), *gltB* M5 (yellow) or *glmS* M 1-3-4-6-7 (blue). Scrambling of L-Gln in mutant lysates confirms that several enzymes contribute glutaminase activity and that single mutants do not sufficiently reduce L-Gln to L-Glu conversion. [^15^N, ^1^H] BEST-TROSY spectra were acquired with CypD samples of 100–150 µM in Na-phosphate, pH 7.0, at 303 K. A19 and M5 spectra were recorded on a 900 MHz, M 1-3-4-6-7 spectrum on a 700 MHz spectrometer, respectively

**Fig. 6:**
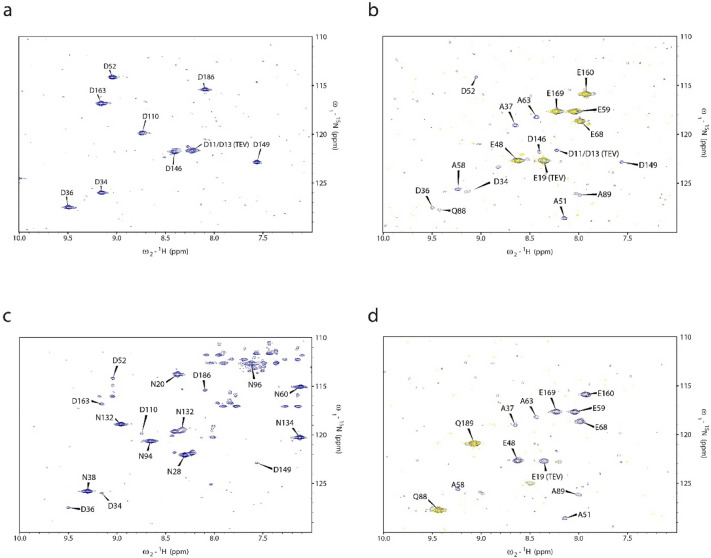
Selective CF labeling of CypD (43–207) with “Stablelabel” S30 lysate with low contour levels. Blue spectra illustrate results of CypD selective labeling with ^15^N L-Asp (**a**), ^15^N L-Glu (**b**), ^15^N L-Asn (**c**) or ^15^N L-Gln (**d**) using “Stablelabel”. The yellow spectrum in **b** was obtained after addition of 20 mM AOA in the CF expression with “Stablelabel” to suppress L-Glu to L-Ala label conversion. The yellow spectrum in **d** was obtained after incubation of “Stablelabel” lysate with 5 mM DON at RT for 1 h before CypD expression. [^15^N, ^1^H] BEST-TROSY spectra were measured on CypD samples of 100–150 µM concentration in sodium phosphate buffer pH 7.0 at 303 K. Spectra were recorded on 600 or 700 MHz spectrometers

“Stablelabel” S30 lysates were compared with A19 S30 lysates prepared at identical conditions regarding ^15^N label stability of L-Asp, L-Asn, L-Glu and L-Gln. The previously assigned chaperone derivative CypD (43–207) was used as model protein. CF synthesis of CypD (43–207) with A19 S30 lysates and ^15^N-L-Asp resulted in 22 signals derived from the 9 L-Asp residues but also from distinct L-Glu, L-Gln and L-Ala residues. In contrast, CF synthesis and labeling of CypD (43–207) with “Stablelabel” S30 lysates resulted only in the 9 expected L-Asp signals (Fig. 4a). The primary step of L-Asp scrambling in A19 S30 lysates is its conversion to L-Glu by AspC. From L-Glu, secondary conversions to L-Ala and L-Gln occur by glutamine synthase (GlnA) and glutamate-pyruvate transaminases (AlaA/AlaC) which explains the lower signal intensities of Q and A. Thus, for ^15^N-L-Asp labeling, AspC is central for increased ambiguity of 2D NMR spectra. The weak scrambling to L-Asn in A19 S30 lysates detectable upon GFP synthesis was also eliminated by asparagine synthase A (AsnA) deletion (Figs. 4a and 5). A similar contribution by AspC was also observed with ^15^N-L-Glu labeling where no L-Asp signals were observed as no conversion from L-Glu to L-Asp was possible. Furthermore, *glnA* inactivation eliminated L-Gln signals from the spectrum (Fig. 4b). Apart from the expected L-Glu residues, only weak signals from L-Ala residues remained in the NMR spectrum obtained with “Stablelabel” S30 lysates (Fig. 4b). As addition of 20 mM aminooxyacetate (AOA) eliminated those signals, they must be due to a PLP dependent transamination reaction potentially catalyzed by residual AlaA/AlaC. Another severe amino acid scrambling with A19 S30 lysates was observed for L-Asn as it is primarily converted to L-Asp by asparaginases A and B (AnsA/AnsB), secondary to L-Glu by AspC and tertiary to L-Gln by GlnA and L-Ala by AlaA/AlaC (Figs. 1 and 4c). The deletion of *ansA*/*ansB* in “Stablelabel” eliminated conversion to L-Asp, abolishing further secondary and tertiary conversions (Fig. 4c).﻿ ﻿Despite numerous mutations, it was not possible to eliminate L-Gln to L-Glu conversion and corresponding 15N-L-Gln scrambling is still noted in “Stablelabel” S30 lysates. Only treatment with 5 mM 6-diazo-5-oxo-L-norleucine (DON) for 1 h at RT under gentle agitation inhibited glutaminase activity in the lysate (Fig. 4d). We identified four remaining enzymes in the A19 S30 lysate potentially catalyzing this reaction: GlmS, GltB, GuaA and AsnB. Single deletions of *gltB* or *glmS* did not yield satisfactory results regarding suppression of L-Gln conversion (Fig. 5). Unfortunately, a cumulative mutant of *gltB* and *glmS* displayed significant growth problems and yielded unproductive S30 lysates. Furthermore, deletions of *guaA* could not be obtained and are thus probably not viable in A19. Deletion of asnB in “Stablelabel” was not further approached as no improvement in label scrambling was expected. The already minimal L-Asp to L-Asn conversion was already abolished by the *asnA* mutation, indicating that residual AsnB does not play a role for this reaction in the S30 lysate. To detect all residual scrambling events, contour levels of spectra from Fig. 4 were set to low threshold values until appearance of background noise (Fig. 6). L-Asp spectra did not show further signals apart from L-Asp (Fig. 6a), while L-Glu showed some additional peaks corresponding to L-Ala entities that disappeared upon addition of AOA during expression (Fig. 6b). Weak signals of L-Asp also appeared in the L-Asn spectrum indicating weak residual asparaginase activity (Fig. 6c). Since both asparaginases in “Stablelabel” were deleted, asparaginase activity is most likely brought into the reaction by T7RNAP that is only partially purified by ion exchange chromatography. Likewise, lowering contour levels in L-Gln spectrum, labeled samples indicated some minor conversion from L-Glu to L-Ala, but also residual glutaminase activity (Fig. 6d).﻿

.

**Fig. 7:**
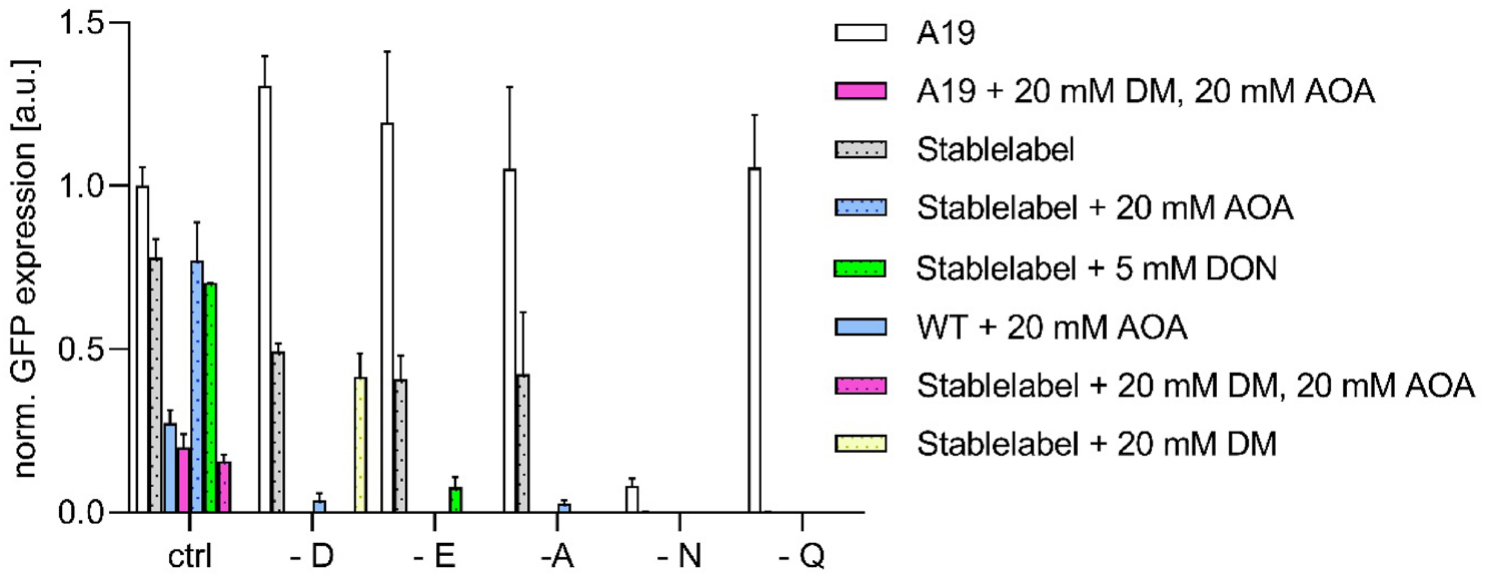
Amino acid synthesis in S30 lysates of either A19 or “Stablelabel”. GFP was CF expressed under standard conditions using the two different lysates either with all amino acids supplied or with L-Ala, L-Asp, L-Asn, L-Glu or L-Gln omitted from the reaction. Expression controls with inhibitors DM + AOA, AOA and DON were included. Control are values of reactions in presence of all 20 amino acids. All data are normalized to the A19 control value (1 = 3.5 mg/mL)

### Amino acid synthesis in “Stablelabel” S30 lysates

To further exploit the potential of “Stablelabel” S30 lysates, we conducted in vitro assays to investigate background amino acid conversions that were not necessarily detectable by increased NMR signal complexity, but would still result in label dilutions. This was carried out by expressing GFP with either A19 or “Stablelabel” S30 lysates in absence of either L-Asp, L-Glu, L-Asn, L-Gln or L-Ala and with or without the use of inhibitors. GFP expression levels were even slightly increased in A19 S30 lysates in absence of L-Asp or L-Glu indicating high synthesis capacity for these amino acids by conversion from other lysate compounds (Figs. 2 and 7).

Despite no detectable amino group transfers from L-Glu to L-Asp by NMR, we found that “Stablelabel” S30 lysates still show ~ 80% of their GFP expression capacity if L-Asp is completely omitted from the CF reaction (Fig. 5). Due to the “Stablelabel” mutations, conversion of L-Asp from L-Glu by AspC or from L-Asn by AnsA/B could be excluded. The assumption that L-Asp might be produced from fumarate by aspartate ammonia lyase (gene: *aspA*) was not confirmed as the specific inhibitor D-malate (DM) showed no effect on GFP expression. Instead, we found that residual L-Asp conversions must be due to PLP dependent transamination reactions as 20 mM AOA almost completely abolished GFP expression without L-Asp (Fig. 7). The promiscuous aminotransferase AraT (gene: *tyrB*) might contribute to this conversion as it also recognizes L-Asp as substrate (Hayashi et al. 1993). However, AraT was not detected in our proteomics analysis and ^15^N labeled amino transfer to aromatic residues was not observed (Fig. 4a–d). The weak scrambling to L-Asn in A19 S30 lysates detectable upon GFP synthesis was also eliminated by asparagine synthase A (*asnA*) deletion (Figs. 4a and 5)

Synthesis of L-Ala in “Stablelabel” S30 lysates was still observed as GFP levels of reactions without L-Ala are similar to those obtained with standard CF expression reactions. Conversions by PLP dependent glutamate-pyruvate aminotransferases (genes: *alaA*/*alaC*) that GFP expression was completely abolished in "Stablelable" lysates when L-Gln was omitted from the expression (Fig. 7). This corresponds to our NMR results (Fig. 4b) and confirms that L-Gln is only produced from L-Glu by glutamine synthase (*glnA*). GFP expression without L-Glu was decreased to ~ 65% of the expression capacity of "Stablelabel" lysates. This agrees with our NMR studies (Fig. 4a, d) and is most likely due to the restricted supply of L-Glu from L-Asp, while L-Glu conversion from L-Gln remains (Fig. 7). However, this pathway can be addressed by treatment of lysate with the inhibitor DON. Application of both inhibitors DON and AOA had no negative effect on GFP yield obtained with “Stablelabel”. DM in combination with AOA however, significantly reduced productivity in lysates of “Stablelabel” as well as of A19 (Fig. 7).

### Methyl labeled precursor conversion

Specific methyl side-chain labeling of larger targets and membrane proteins is highly useful as the threefold proton multiplicity of the labeled methyl group increases NMR signal sensitivity. Thereby, the methyl group clustering in protein cores can provide valuable information on structural restraints via NOE measurements. In previous work, we showed the precursor-based selective methyl labeling of CF synthesized proteins by taking advantage of the promiscuous IlvE enzyme present in standard A19 S30 lysates (Lazarova et al. 2018). However, efficient KIV to L-Val conversion directly in the CF reaction was only obtained by some extra supply of purified IlvE. Unfortunately, addition of aminotransferase IlvE into CF labeling reactions could cause undesired amino acid scrambling as side effect. The elimination of IlvE in “Stablelabel” S30 lysates in concert with the exemplified pre-conversion of KIV or MOV precursors could thus be a useful strategy for a better controlled methyl side-chain labeling in combination with other labeling strategies (Lazarova et al. 2018).

**Fig. 8:**
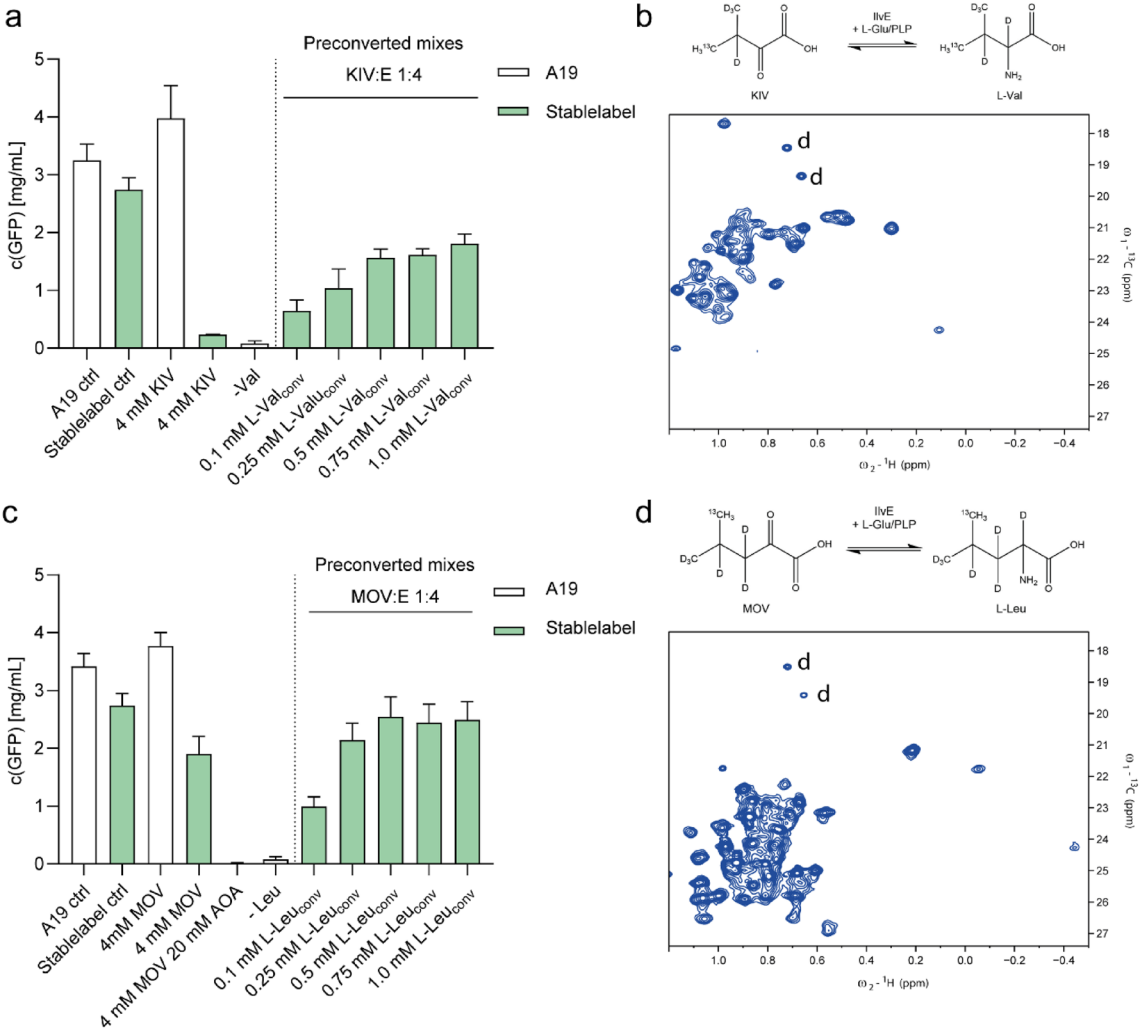
Conversion of methyl labeled precursor KIV to L-Val and MOV to L-Leu. GFP expression was carried out either with A19 (white bars) or with “Stablelabel” (green bars) S30 lysates. **a**/**c** The first two bars indicate control expressions with both lysates with 1 mM L-Val/L-Leu in the reaction. The next two bars show GFP expression with L-Val/L-Leu replaced by 4 mM KIV/MOV. For MOV, the same reaction was carried out in presence of 20 mM AOA, an unselective transaminase inhibitor. The next bar (-Val/-Leu) indicates expression with A19 S30 lysate without L-Val/L-Leu. The next section of the graph shows GFP expression with L-Val/L-Leu replaced by converted KIV/L-Glu or MOV/L-Glu mixtures. The indicated molarities represent the theoretical L-Val/L-Leu concentration if all KIV/MOV was converted. **b** [^13^C, ^1^H] XL-ALSOFAST-HMQC spectrum of PR in non-deuterated diC7PC labeled with L-Val (4-^13^C, 2, 3, 4, 4, 4,-D5) converted from KIV (3-methyl-^13^C, 3, 4, 4, 4-D4). Detergent signals are labeled with a “d” **d** [^13^C, ^1^H] XL-ALSOFAST-HMQC spectrum of PR in non-deuterated diC7PC labeled with L-Leu (5-^13^C, 2, 3, 3, 4, 5, 5, 5-D7) converted from MOV (4-methyl-^13^C, 3, 3, 4, 5, 5, 5-D6). Spectra were acquired on a 950 MHz Bruker Avance Neo spectrometer at a temperature of 313 K. Conversion of both precursors was carried out in ~ 90% deuterated buffer to avoid proton incorporation at Cα during transamination by IlvE

Initial GFP control reactions with “Stablelabel” S30 lysates and in absence of L-Val verified that the *ilvE* deletion almost eliminated the conversion of KIV to L-Val. In contrast, GFP expression in L-Val devoid A19 S30 lysates was hardly affected (Fig. 8a). This also indicates that the abundance of another potential scrambling enzyme, valine-pyruvate aminotransferase AvtA, is rather low in “Stablelabel” S30 lysates. In case of MOV to L-Leu conversion, we found that “Stablelabel” S30 lysates were still reaching ~ 70% GFP synthesis of corresponding controls. Addition of 20 mM AOA eliminated this conversion, indicating a certain aminotransferase activity that might be attributed to minute amounts of AraT (Fig. 8b).

**Fig. 9:**
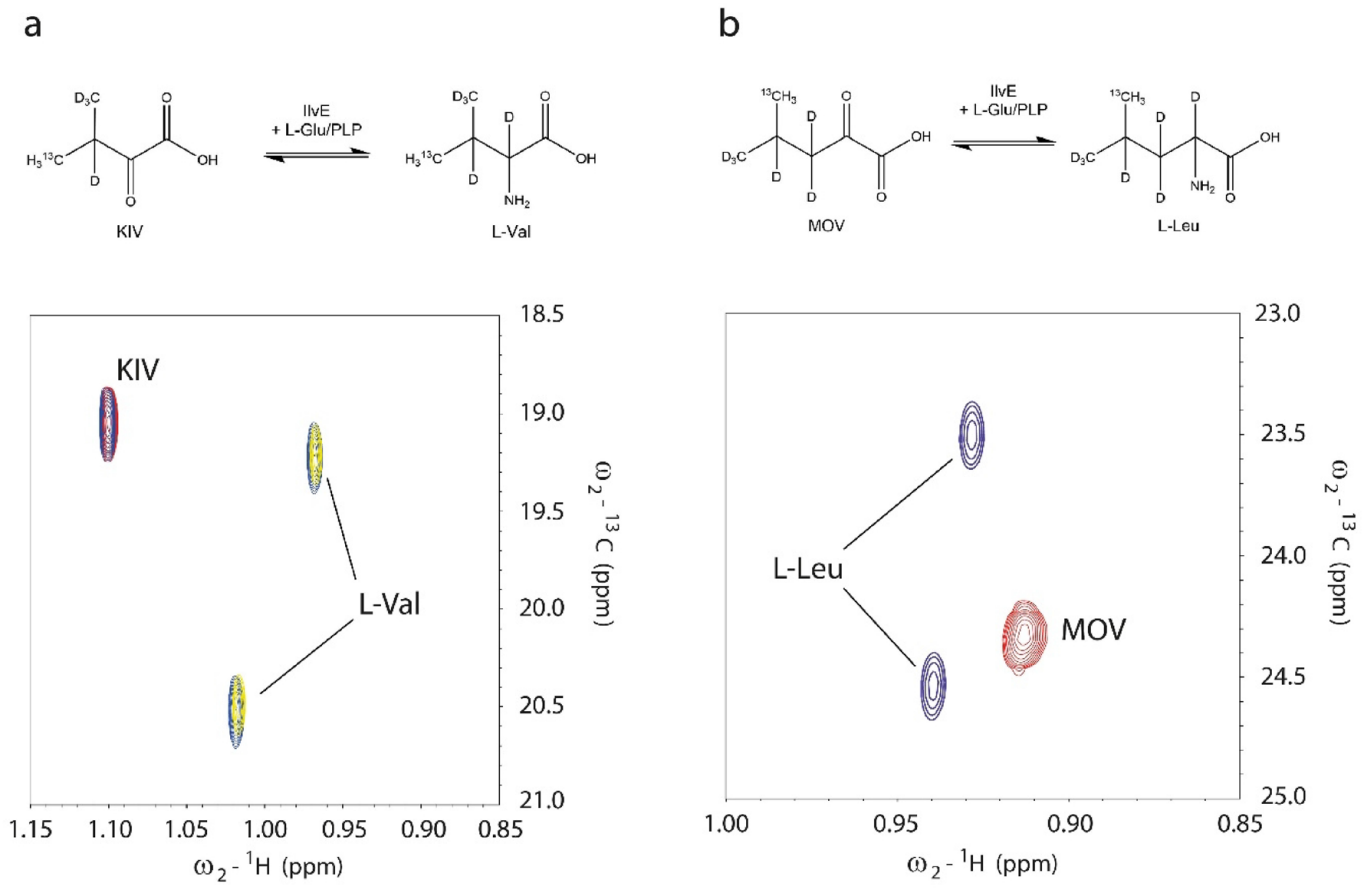
Conversion of KIV (3-methyl-^13^C, 3, 4, 4, 4-D4) to L-Val (3-methyl-^13^C, 2, 3, 4, 4, 4,-D5) and MOV (4-methyl-^13^C, 3, 3, 4, 5, 5, 5-D6) to L-Leu (4-methyl-^13^C, 2, 3, 3, 4, 5, 5, 5-D7) precursors by supplied IlvE. **a** Overlay of non-converted KIV (red), KIV after conversion with 75 mM KIV and 75 mM L-Glu (blue) and KIV after conversion with 75 mM KIV and 300 mM L-Glu (yellow). **b** Overlay of non-converted MOV (red) and MOV after conversion with 24 mM MOV and 100 mM L-Glu (blue). [^13^C, ^1^H]-HSQC spectra were recorded at T = 313 K on a Bruker Avance II 500 MHz spectrometer, equipped with a room-temperature ^1^H{^13^C/^15^N} three-axes gradient triple-resonance probe. Precursor conversion was performed in 100 mM Tris–HCl, pH 8.0, 100 mM NaCl dissolved in D_2_O and 1 mg/mL IlvE. Upon completion of conversion, IlvE was removed by ultrafiltration and the mixtures were diluted 1:50 in 25 mM Na-acetate, pH 5.0 containing 5% D_2_O and 0.15 mM DSS. Non converted precursors were measured under the same conditions

We next approached the KIV and MOV precursor conversion in separate controlled reactions and the subsequent supply of the resulting amino acids to the CF reaction. Conversion of the precursors KIV to L-Val and MOV to L-Leu by IlvE was first performed in 10 mL reactions using 1 mg/mL purified recombinant IlvE and equimolar concentrations of the precursors and of the amino group donor L-Glu. Conversion reactions were incubated for 16 h at 30 °C and IlvE was then subsequently removed by ultrafiltration. NMR control experiments conducted with the conversion mixtures showed that four-fold excess of the amino group donor L-Glu favors the almost complete conversion of KIV and MOV to the respective amino acids (Fig. 9). Various volumes of the filtrated conversion reaction were added to CF expression reactions of GFP in absence of L-Val. Assuming that all KIV is converted to L-Val, we found that a final concentration corresponding to 0.5 mM converted L-Val in the CF reaction was sufficient to restore the standard productivity of “Stablelabel” S30 lysates for GFP synthesis (Fig. 8a). In the case of MOV, we determined a final concentration corresponding to 0.25 mM converted L-Leu as being sufficient to fully restore GFP synthesis. This however, might require further adjustment when working with other protein targets.

As model for a larger membrane protein, we selected the 27.6 kDa seven transmembrane domain containing light-activated proton pump proteorhodopsin (PR). The protein was CF synthesized in “Stablelabel” S30 lysates and cotranslationally solubilized in presence of a supplied mixture of 0.1% diC7PC and 0.4% GDN (Reckel et al. 2011). For the preparation of a L-Leu methyl-selective labeled NMR sample, we adjusted an estimated final reaction concentration of 0.5 mM L-Leu (5-^13^C, 2, 3, 3, 4, 5, 5, 5-D7) with the supplied MOV (4-methyl-^13^C, 3, 3, 4, 5, 5, 5-D6) conversion mixture. Furthermore, 20 mM AOA were added to the CF expression reaction to avoid potential label dilution by the observed MOV dependent residual L-Leu synthesis. For L-Val labeling converted from KIV (3-methyl-^13^C, 3, 4, 4, 4-D4), no AOA was added, since only IlvE contributes to KIV conversion in A19 S30 lysates, while it is eliminated in “Stablelabel” S30 lysates (Fig. 8a).

[^13^C, ^1^H] XL-ALSOFAST-HMQC spectra of CF synthesized PR in diC7PC labeled with converted L-Val (4-^13^C, 2, 3, 4, 4, 4,-D5) (Fig. 8b) and L-Leu (5-^13^C, 2, 3, 3, 4, 5, 5, 5-D7) (Fig. 8d) demonstrate the application of “Stablelabel” S30 lysates for selective methyl group labeling of membrane proteins. Since either the pro-S or the pro-R [^13^C, ^1^H] methyl group in L-Val (4-^13^C, 2, 3, 4, 4, 4,-D5) is incorporated in a non-stereospecific manner, ~ 37 detected peaks are in the expected range of 40 potential L-Val (4-^13^C, 2, 3, 4, 4, 4,-D5) signals. The PR spectrum with converted KIV (3-methyl-^13^C, 3, 4, 4, 4-D4) is in accordance with data previously acquired with KIV (3-methyl-^13^C, 3, 4, 4, 4-D4) conversion directly in the CF reaction with A19 S30 lysates containing a combination of residual and supplied IlvE (Lazarova et al. 2018). The relatively broad signals are due to the use of non-deuterated diC7PC for PR solubilization in addition to its synthesis with non-deuterated amino acids (Fig. 8b). Herein, we also show the first NMR spectrum of a membrane protein CF labeled with L-Leu (5-^13^C, 2, 3, 3, 4, 5, 5, 5-D7) enzymatically converted from MOV (4-methyl-^13^C, 3, 3, 4, 5, 5, 5-D6) by the documented strategy (Fig. 8d). The number of signals is difficult to estimate due to strong signal overlap. PR contains 27 L-Leu residues, but the [^13^C, ^1^H] methyl groups were not stereospecifically labeled in the MOV (4-methyl-^13^C, 3, 3, 4, 5, 5, 5-D6) precursor, as it was the case with the KIV experiment.

## Discussion

The open accessible CF system allows the efficient selective and combinatorial labeling of proteins. Despite reduced scrambling activity in processed S30 lysates, several enzymatic conversions still cause significant problems. Supplying inhibitor cocktails or using the more defined PURE system are strategies to suppress label conversion, while increased costs and lower productivity might be considered (Lavickova and Maerkl 2019; Kuruma and Ueda 2015). This study describes a new alternative approach by implementing an engineered derivative of the *E. coli* K12 strain A19, known to yield highly productive CF S30 lysates. The proteome background of A19 S30 lysates prepared by standardized protocols has been determined and the revealed data support the CF synthesis of proteins in better controlled conditions (Foshag et al. 2018). S30 lysates obtained from the engineered “Stablelabel” strain (DSM 116065) will thus further help to streamline the workflow for CF protein labelling and eliminate the requirement for inhibitors in several applications. This will be particularly useful for selective and combinatorial labeling approaches (Laguerre et al. 2015; Löhr et al. 2019).

Unfortunately, not all identified genes responsible for residual NMR label scrambling activity in standardized A19 S30 lysates could be addressed by mutagenesis successfully. Various attempts to delete *guaA* or *alaC* failed, presumably due to strong effects on A19 cell viability. Furthermore, at least four detected enzymes are probably involved in the glutaminase activity of A19 S30 lysates. According to the previously determined emPAI values, GltB, GlmS and GuaA were most prominent in addition to minor fractions of AsnB (Foshag et al. 2018). A19 *glmS* mutants lost viability if grown in presence of glucose and they further required the supply of glucosamine or GlcNAc for cell growth. This is in accordance with previous observations reporting the transition of *glmS* mutants from rods to spheroplasts if grown on glucose and requiring glucosamine for growth (Wu and Wu 1971). It was suggested that glucose impaired glucosamine uptake by the phosphotransferase systems (Kundig et al. 1964). As expected, single mutants of *gltB* or *glmS* did not notably reduce scrambling in corresponding S30 lysates and cumulative *gltB* and *glmS* mutants already showed restricted cell proliferation capability. Considering the additional failure to inactivate *guaA*, it was therefore decided to leave all four genes putatively involved in L-Gln scrambling intact. This avoided the requirement of growth media modifications as well as negative effects on “Stablelabel” cell viability and its S30 lysate efficiency. Epitope tagging by genetically adding a 6×His-tag or similar purification tags to these enzymes might be considered as alternative strategy to remove glutaminase activity posttranslational from CF lysates. However, our approach to address GlnA removal using λ-phage based recombination, did not work out as already the genetic *glnA* modification resulted in absence of glutamine synthetase activity in the final S30 lysate. It can therefore be speculated that the attached 6×His-tag to GlnA resulted in inactive or instable enzyme. It is also possible that the 32 bp FRT cloning scar remaining after marker recovery abolished *glnA* expression. Recombination via CRISPR-Cas9 or other scarless recombination techniques might thus be considered in future (Datsenko and Wanner 2000; Stringer et al. 2012; Ronda et al. 2016). Weak L-Glu to L-Ala scrambling was still present but can be addressed by addition of AOA, an unspecific transaminase inhibitor. A valuable benefit of “Stablelabel” lysates is the reduction of asparaginase activity by ansA/B deletion that would otherwise require the limited available inhibitor 5-diazo-4-oxo-L-norvaline. It is important to note that CF S30 lysates originating from different *E. coli* strains might show considerable variations in their scrambling backgrounds. A19 S30 lysates show inherently low aspartate lyase activity converting L-Asp to L-Asn, while the production of L-Ala from other compounds is relatively high (Yokoyama et al. 2011). We nevertheless deleted *asnA* in “Stablelabel” to ensure the absence of L-Asp to L-Asn scrambling also after potential future protocol modifications, such as change of cultivation or CF conditions.

Solution NMR spectroscopy of side-chain methyl groups of L-Val, L-Leu and L-Ile was addressed by diverse synthesis strategies that allowed selective modification of side chains with unique labeling patterns (Lichtenecker et al. 2004; Tugarinov and Kay 2003; Gardner and Kay 1997; Schütz and Sprangers 2020). This paved the way for a variety of cell-based and CF labeling strategies of proteins (Lazarova et al. 2018; Gans et al. 2010; Kerfah et al. 2015; Törner et al. 2021; Linser et al. 2014). We present the first report on methyl side-chain labeling of an integral membrane protein by converting the precursors KIV and MOV in separate reactions. The deletion of *ilvE* in “Stablelabel” reduces L-Leu and L-Val instability. The demonstrated strategy to preconvert specifically labeled derivatives of the precursors KIV and MOV into L-Val and L-Leu provides better control over reaction equilibrium and conversion efficiency, if compared with conversion in A19 S30 lysates catalyzed by residual IlvE enzyme (Lichtenecker et al. 2015). Furthermore, deuterium/proton back exchange at the Cα position is omitted if precursor conversion is carried out in deuterated environment. 10–15 mg of the respective precursor is sufficient to obtain 600 µL of a 100 µM PR sample suitable for NMR analysis. At current prices of 1366 $/g for KIV (3-methyl-^13^C, 3, 4, 4, 4-D4), this corresponds to ~ 20 $ per sample. The required amount of KIV is thus only approx. 5–10% of the amount needed for 1 L *E. coli* culture based expression, while this certainly will also depend on the individual expression yields. Unfortunately, MOV (4-methyl-^13^C, 3, 3, 4, 5, 5, 5-D6) is commercially unavailable. Nevertheless, NMR spectra of protein samples labeled with either L-Val (4-^13^C, 2, 3, 4, 4, 4,-D5) or L-Leu (5-^13^C, 2, 3, 3, 4, 5, 5, 5-D7) but lacking stereospecifity of the ^13^C methyl groups will still remain more complex if compared to the use of SAIL amino acids (Kainosho et al. 2006).

Solubilization in detergent is the current standard for NMR analysis of membrane proteins and even large and complex G-protein coupled receptors can be addressed as demonstrated with the β_1_ adrenergic receptor receptor (Isogai et al. 2016; Grahl et al. 2020; Rößler et al. 2020b). L-Val, L-Leu and L-Ile are common residues in membrane protein cores and they are frequently involved in the formation of specific orthosteric ligand binding pockets (Yang et al. 2021; Venkatakrishnan et al. 2013). NMR analysis of selectively labeled samples could therefore reveal important insights in G-protein coupled receptor dynamics and ligand interaction (Isogai et al. 2016; Grahl et al. 2020; Rößler et al. 2020b; Krug et al. 2020). Recent reports demonstrated the potential of CF expression platforms to prepare samples of full-length G-protein coupled receptors in sufficient yields and quality to be suitable for structural NMR analysis (Krug et al. 2020; Pacull et al. 2020). “Stablelabel” S30 lysates in combination with the high versatility and advanced labeling tools of CF synthesis could thus further support sample production pipelines for NMR approaches with membrane proteins. Future refinements might rather focus on membrane protein samples inserted into more native-like lipid environments of nanodiscs and similar nanoparticles. Implementation of engineered nanodiscs combined with deuteration could help address the intrinsically slow tumbling rates of large membrane protein/nanoparticle complexes (Hagn et al. 2018; Puthenveetil et al. 2017). A straightforward strategy to prepare such samples could be by cotranslational insertion of CF synthesized membrane proteins directly into membranes of supplied preformed nanodiscs (Levin et al. 2020; Köck et al. 2022). However, the systematic evaluation of a suitable lipid composition of the nanodisc membrane, supply of stabilizing ligands into the CF reaction and proper selection of the membrane insertion strategy are essential to obtain high quality samples (Köck et al. 2022; Levin et al. 2022).

## Conclusion

The CF generation of NMR samples requires a versatile and continuously growing toolbox to optimize labeling schemes and to overcome problems with non-desired side effects. The constructed A19 “Stablelabel” derivative and resulting S30 lysates add a new feature to CF expression platforms further facilitating specific protein labeling options. For ^15^N labeling of L-Asp and L-Asn, no additional inhibitors are required. Labeling of L-Glu is achieved by suppressing weak scrambling to L-Ala by addition of AOA and L-Gln to L-Glu scrambling can be addressed by lysate treatment with DON. In addition, the presented application of controlled enzymatic L-Val and L-Leu conversion from specifically methyl labeled precursors could help to address the NMR analysis of even larger membrane proteins in complex hydrophobic environments.
